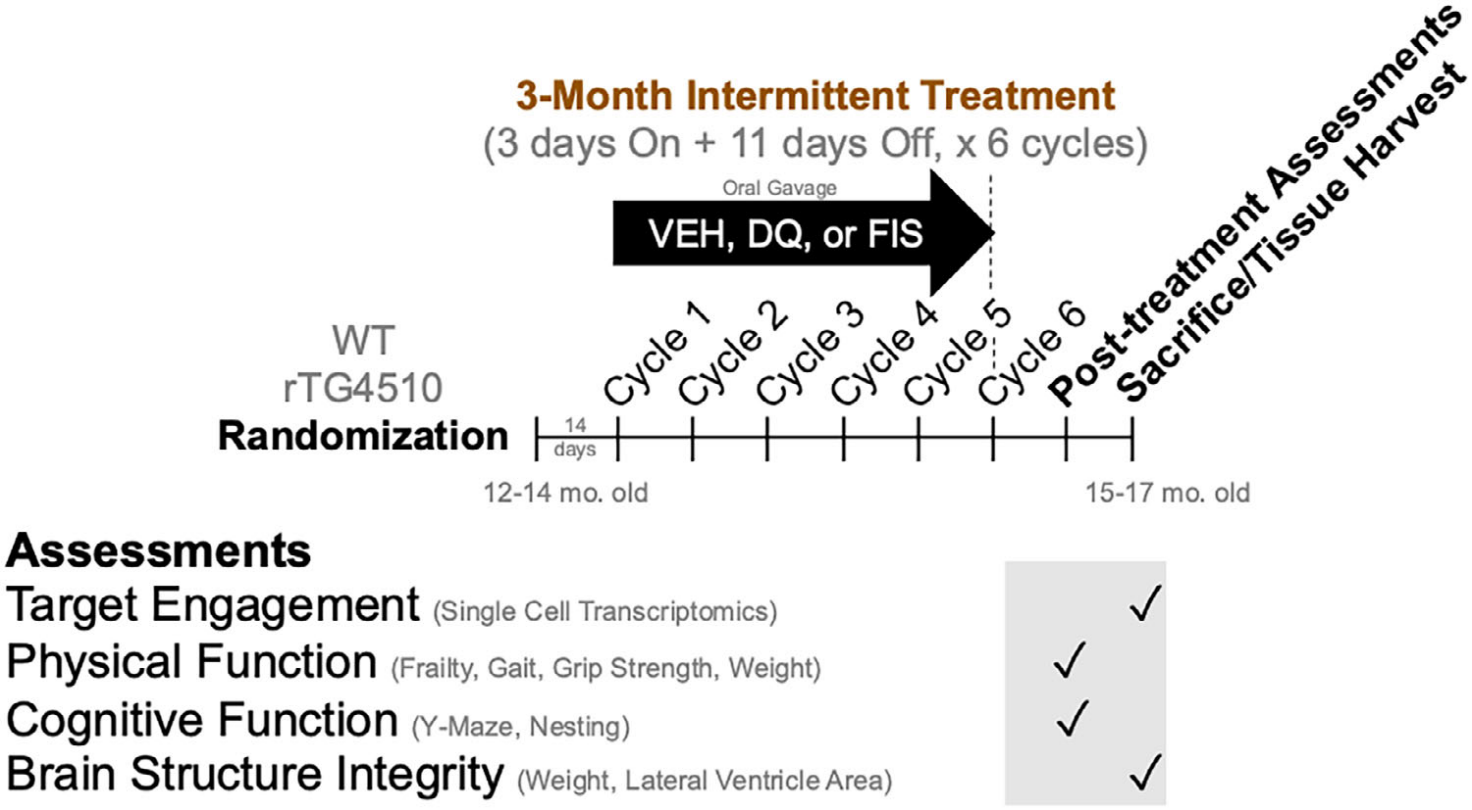# Head‐to‐head comparison of two senolytic therapies in a mouse model of tauopathy

**DOI:** 10.1002/alz70855_104947

**Published:** 2025-12-24

**Authors:** Valentina R. Garbarino, Timothy C. Orr, Miranda E. Orr

**Affiliations:** ^1^ Glenn Biggs Institute for Alzheimer's & Neurodegenerative Diseases, San Antonio, TX, USA; ^2^ University of Texas Health San Antonio, San Antonio, TX, USA; ^3^ Washington University School of Medicine, St. Louis, MO, USA

## Abstract

**Background:**

Tau protein accumulation induces cellular senescence in Alzheimer's disease (AD). Treating tau transgenic mice with the senolytic therapy, dasatinib plus quercetin (DQ), to clear senescent cells reduced brain pathology and improved structure and function. DQ has since been moved into clinical AD trials. Herein, we report results from a direct comparison of DQ to fisetin FIS, a senolytic with a potentially more favorable side‐effect profile.

**Method:**

Fourteen‐month‐old female and male wild type (WT) and tau transgenic (rTg4510) mice received 12 weeks of intermittent DQ, FIS or vehicle by oral gavage. Physical and cognitive assessments, and RNA sequencing analysis on brain tissue were performed.

**Result:**

Both senolytics significantly increased frailty in female and male rTg4510 mice (*p* < 0.05). There was no effect of senolytic on working memory measured with Y‐Maze, or hippocampal dependent nest building behavior in rTg4510 mice. Spontaneous alterations percentage increased in WT male mice treated with FIS. Both senolytics significantly improved nest building in WT females (*p* < 0.02). Grip duration was reduced by both senolytics in WT and rTg4510 female mice and by FIS in WT and rTg4510 treated males compared to vehicle treated controls (*p* < 0.03). FIS was protective against ventricular enlargement, indicative of neurodegeneration in rTg4510 females. RNA analysis revealed differential expression of 232 genes with senolytic treatment; with an overlap of 54 genes that were differentially expressed by both treatments.

**Conclusion:**

Outcomes related to physical and cognitive function were largely unchanged by senolytic in rTg4510 mice, indicating our model may have been too advanced by the time of drug initiation to see benefits from the treatment. Subtle, functional changes observed after senolytic in WT mice and the RNA sequencing data highlight the need for additional studies to evaluate the appropriateness of selected senolytic, dose, and initiation timeline, for use in AD relevant populations.